# Relationship between client laughter and session outcomes in metaverse counseling

**DOI:** 10.1186/s40359-024-02260-0

**Published:** 2024-12-18

**Authors:** Jieun Kang, Woo Hyun Baek, Yeon Bin Jeong, Hyerin Yang, Seongchan Lee, Sang Min Lee

**Affiliations:** 1 Department of Education, Korea University, Seoul, South Korea; 2 YATAV, Inc, Seoul, South Korea

**Keywords:** Metaverse counseling, Avatar, Virtual psychotherapy, Client laughter, Session outcomes

## Abstract

Considering the growing interest in VR psychotherapy, this study investigated the relationship between client laughter and session outcomes in metaverse counseling. To investigate the relationships between types of client laughter and session outcomes in metaverse counseling, we employed a multilevel analysis by separating the variables into two levels: session-level (between-sessions) and client-level (between-clients). The dataset included 159 sessions nested among 26 clients. This study found that clients’ cheerful and nervous laughter positively impacted session outcomes at the session level (within individual clients). However, when considering client-level laughter events (between-client), nervous laughter at the session level was not significantly related to session outcomes. Polite, reflective, and contemptuous laughter showed no significant relationship with the session outcomes. None of the laughter events were related to session outcomes at the client level (between clients). However, there was a significant within-level interaction effect between session and cheerful laughter on session outcomes. The implications of the effects of client laughter are discussed in metaverse counseling by comparing them with those of in-person counseling.

## Relationships between Client Laughter and Session Outcome in Metaverse Counseling

In recent years, the integration of virtual reality (VR) and the metaverse has steered transformative changes across various aspects of our lives, impacting our modes of communication, work, and social interaction [20]. These advancements have redefined our daily experiences and introduced notable shifts within the realm of mental health care through the introduction of metaverse counseling [7]. This groundbreaking therapeutic approach capitalizes on the immersive and interactive capabilities of virtual reality to provide support and interventions for individuals confronting a spectrum of mental health challenges [8]. In South Korea, metaverse counseling has gained significant traction in elementary and secondary schools, universities, and Employee Assistance Programs. Notably, 141 educational institutions have adopted the metaverse counseling platform, aptly named “MetaForest” (see https://youtu.be/Y8xkKOyipoM). This platform, which has over 10,000 students, has become a vital hub for delivering counseling services, underscoring the widespread acceptance and utilization of this innovative approach [23].

Metaverse counseling broadens the scope of traditional in-person therapy by immersing clients and therapists in a virtual setting, enabling them to engage in and tackle emotional and psychological challenges. Cho et al. [8] explored the outcomes of counseling in metaverse and traditional in-person modalities. Their findings indicated that metaverse counseling resulted in more noticeable improvements in specific aspects of psychological symptoms, such as depression and anxiety, than in-person counseling. Furthermore, the study reported a higher level of therapeutic rapport and client satisfaction with the metaverse modality than with traditional face-to-face therapy. These results suggest a potential inclination toward establishing a more robust working alliance between counselors and clients at an accelerated pace within the metaverse counseling framework.

Although Cho et al. [8] demonstrated the usefulness and effectiveness of metaverse counseling, there remains a significant gap in our understanding of the factors that influence the effectiveness of this therapeutic approach. An essential aspect that requires further exploration within the domain of metaverse counseling is the non-verbal behaviors exhibited by clients during therapy sessions. Within the metaverse counseling platform MetaForest, cutting-edge deep learning technology is employed to identify and interpret facial expressions (see metaforest.us). This technology enables avatars to mirror the real facial expressions of the therapist and the client. Consequently, this capability allows for the interpretation of non-verbal behaviors, marking a notable advancement in the provision of metaverse counseling services.

Previous studies have produced mixed findings on whether avatars’ non-verbal cues in virtual environments resemble real-life interactions, reflecting the complexity of translating social signals into digital spaces [32]. Some researchers have reported that digital avatars often fail to capture the full range of non-verbal cues (e.g., micro-expressions, subtle shifts in body language, complex facial expressions) [11]. Yet, other studies suggest that avatars' non-verbal cues can emulate real-life social interactions effectively, depending on the therapeutic presence or immersion level [43]. This may especially vary depending on the context and the counseling medium used; therefore specifically, this study tried to focus on the impact of non-verbal expressions within metaverse counseling and how these might exhibit different dynamics compared to real-life counseling interactions.

Among various client non-verbal behaviors, laughter emerges as a critical element, as it possesses the unique potential to empower therapists to enhance their proficiency in forging a robust therapeutic alliance, playing a pivotal role in advancing and optimizing counseling outcomes [14]. Laughter can be part of the therapeutic process. Laughter may mirror the mechanisms underlying spoken communication and serve as an indicator of the effectiveness of therapeutic exchange. Occasionally, client laughter can affect the counseling process, such as when a client has a big emotional release or when a client uses laughter to hide their genuine emotions. Clients can use laughter in unique and creative ways. Therapists must be careful because of the risks, especially when clients use dark or insensitive laughter [5].

Numerous scholars, including Gupta [14], Marci et al. [33], and Nelson [41], have conducted empirical research to examine the significance of client laughter in the therapeutic process and its outcomes. Nevertheless, the specific dynamics of client laughter in a metaverse counseling environment are yet to be explored. This study aims to uncover the role of client laughter and its ramifications in the therapeutic process in the context of metaverse counseling.

## Empirical Studies on Client Laughter and Session Outcomes

Gervaize et al. [12], Falk and Hill [9], Marci et al. [33], and Gupta [14] investigated client laughter in the context of psychotherapy. First, Gervaize et al. [12] suggest risky therapist behaviors can induce strong laughter. Consequently, Falk and Hill [9] critically reviewed and expanded Gervaize et al.’s [12] research by investigating the causes of client laughter in psychotherapy. Their study found that humorous therapist interventions were more effective in eliciting laughter than risky therapist behaviors. Specific types of humor intervention, such as “other humor” and “release of tension,” played a significant role in client laughter. Marci et al. [33] explored the impact of client laughter in psychotherapy by assessing skin conductivity, a measure of electrical activity in sweat glands. They observed elevated skin conductivity during laughter episodes and even more so when the therapist and the client shared laughter, highlighting the significance of laughter in shaping interpersonal dynamics during therapy.

Gupta [14] provided a framework for categorizing laughter characteristics based on observations of laughter episodes from various cases. Gupta focused on the observable verbal and non-verbal aspects of laughter, such as the number of “ha” sounds and duration, rather than the laughter’s function, like its role in establishing a therapeutic relationship. Laughter has five distinct characteristics: (a) cheerful/happy, (b) polite, (c) reflective, (d) contemptuous, and (e) nervous. Each of these characteristics was assessed on a 5-point scale ranging from 1 (no presence) to 5 (strong presence). This classification system allows for a more nuanced analysis of laughter in a therapeutic context. Gupta employed a classification system to investigate the relationship between five distinct types of laughter and session outcomes. Gupta found that sessions characterized by an abundance of reflective laughter received more positive evaluations from clients. Additionally, therapists whose clients exhibited higher levels of reflective laughter tended to receive more favorable session evaluations from these clients. Interestingly, among the therapists’ caseloads, clients displaying the most nervous and contemptuous laughter reported the highest client-rated session evaluations.

## Purpose of Study

We carefully analyzed the therapy sessions conducted in the metaverse to explore different types of client laughter within metaverse counseling based on Gupta’s [14] classification system. During the coding process, four independent judges conducted initial assessments of the client laughter events, followed by collaborative discussions to reach a consensus. As recommended by Hill [17], this approach is deemed more valid than independent ratings because it involves clinical intuition, reasoning, and multiple perspectives. Subsequently, we compared our findings with those of Gupta [14] to identify the specific characteristics and considerations of laughter in the digital context. Finally, employing a multilevel analysis, we will investigate the relationships between the types of client laughter and session outcomes, specifically session satisfaction, in metaverse counseling. This analysis distinguishes between session-level variations in client laughter across sessions and client-level variations in client laughter. This comprehensive examination of client laughter in metaverse counseling aims to enhance the understanding and effectiveness of this emerging counseling approach and facilitate its integration into mainstream therapeutic practices.

## Method

### Participants

#### Clients

This study involved 26 clients (24 females and 2 males; university students), all of whom completed the study without dropping out. Each participant was Korean, representing the racial and ethnic identities of the local population. For privacy and anonymity in metaverse counseling, each client used a nickname, and the counselors did not have access to the participants’ real information. The clients’ personal data, including their student identification number, age, grade, and contact details, remained confidential unless overtly shared with the counselor during the session.

#### Counselors

Seven (six female, one male) master’s and doctoral counselors at a metaverse counseling center participated in this study. The number of clients served by counselors employed at the metaverse counseling center varied from one to eight. The counselors ranged in age from 23 to 33 years (*M* = 27.57, *SD* = 3.78). Additionally, 28.6% had a doctoral degree and an average of 2.09 years of clinical experience (*SD* = 1.82). Every counselor was required to attend monthly case conferences, receive individual clinical supervision, and participate in weekly small-group peer supervision sessions.

#### Coders

Four undergraduate students (two male and two female) majoring in psychology with a fundamental understanding of counseling served as coders. The work of these coders was supervised during each session by two professors specializing in counseling and one doctoral student in the field of counseling. After learning the coding standards as a team, they were divided into two teams (one male and one female per team) and proceeded to code each counseling session.

### Procedures

This study was approved by the Institutional Review Board (IRB Number: 2022–0010) affiliated with the authors’ academic institution. Potential research participants were recruited at a metaverse counseling center from August 2022 to June 2023. The data was collected using a secure digital platform. Before the counseling sessions began, participants received an overarching research introduction and were asked to complete a consent form, ensuring they were informed about the study.

#### Intake and screening

Clients were recruited through Internet discoveries, and their consent and eligibility were determined through a screening questionnaire. During this screening phase, the severity of symptom factors, such as intensity and frequency, and the content of primary psychological concerns were considered to make suitable counselor assignments. Several mental health issues have been reported, including problems related to interpersonal relationships, depression, anxiety, career-related challenges, and academic stress. Clients were provided with comprehensive instructions on how to use the “MetaForest” counseling program, covering aspects such as accessing the platform, using it on their devices, and providing feedback about their counseling experiences through a secure online survey. While counselors were advised to complete therapy within five sessions owing to the policy of the metaverse counseling center, they had the flexibility to extend counseling as needed. On average, metaverse counseling consisted of approximately 6.1 sessions (*SD* = 2.29), with each session lasting approximately 50 min.

#### Counseling sessions

For supervision and coding, all counseling sessions were recorded for approximately 50 min each. When presented to the coders, each session was assigned a specific code to ensure anonymity and confidentiality.

#### Training and coding measures

The coders viewed the metaverse counseling videos and analyzed two aspects: the duration of laughter lasting over three seconds, laughter frequency throughout the entire counseling session, and the level of satisfaction in each session. Before commencing the actual coding process based on the metaverse counseling videos, the coders engaged in discussions and training regarding the analysis of laughter lasting over three seconds and categorized these instances into five specific categories. Each coder underwent training to achieve an intraclass correlation coefficient (ICC) of 0.7 or higher, indicating a strong level of agreement among the raters. A high level of agreement was constantly supervised in each session to be maintained throughout coding, and they were also given a manual for reference. Additionally, the coders assessed the interaction dynamics between the clients and counselors during the sessions. After the four assessors coded the categories as each team, they were divided into two pairs, and the variables were collectively re-evaluated until a consensus was reached. In this process, we adhered to Gupta’s [14] guidelines, which emphasize that consensus judgments involving clinical intuition, reasoning, and various perspectives are considered more reliable than independent ratings.

### Metaverse Counseling Platform

This research utilized a specially designed metaverse counseling platform named “MetaForest” for conducting counseling sessions through avatars in a virtual setting. MetaForest offers several unique features within the counseling environment. These features include facial recognition, motion gestures, themed spaces for counseling, voice modulation, password-protected private counseling areas within the metaverse, and screen sharing. One of the standout features of the platform is its use of advanced deep learning technology to recognize and replicate the real facial expressions of counselors and clients through their avatars. This technology allows for the interpretation of non-verbal cues while preserving the confidentiality of the counseling process. Additionally, MetaForest enables non-verbal interactions through gesture buttons, allowing avatars to perform actions such as greetings, nodding, and raising hands. The platform also provides various themed spaces tailored to different types of counseling sessions, such as individual counseling, group counseling, conferences, and healing gardens, ensuring that the environment matches the specific purpose of each counseling session. To enhance privacy and security during counseling, MetaForest incorporates voice filtering and password protection. MetaForest is accessible on both computers and mobile devices, making it a versatile cross-platform application. More in-depth information on the MetaForest can be found in Cho et al. [7] and Cho et al. [8].

### Measures

#### Laughter characteristics

Gupta [14] focused on identifying and categorizing different laughter characteristics based on a review of existing literature and refined them by observing laughter episodes from various counseling cases. These characteristics were categorized into five types—cheerfulness, politeness, reflectiveness, contemptuousness, and nervousness—each rated on a 5-point scale to assess their presence (i.e. intensity) in counseling interactions.

1. Cheerfulness: Cheerfulness is characterized by mutual enjoyment and awareness of the laughter context between the client and therapist. It is often marked by non-verbal cues such as smiles, relaxed body posture, and facial muscle movements associated with genuine smiles. This is more observed when clients genuinely describe pleasant feelings than when they superficially express pleasure, which is contrary to their inner self.
2. Politeness: Politeness in laughter is related to brief, low-energy laughter that often occurs during pleasantness or small talk. It is characterized by a lack of intense non-verbal cues, such as wrinkles in the skin around the eyes, and is common at the beginning of sessions.
3. Reflectiveness: Reflective laughter is associated with the verbal cues of pondering, thinking, or exploring in the counseling context. Tones are often philosophical and offer new insights or perspectives. Non-verbal cues included steady eye contact, relaxed body posture, a pensive voice tone, and congruence between verbal and non-verbal cues.
4. Contemptuousness: Contemptuous laughter is marked by verbal cues expressing hostility or disapproval toward oneself or others. Non-verbal cues include sighing, scoffing, and physical signs of anger or withdrawal.
5. Nervousness: Nervous laughter occurs when there is incongruence between the content of the discussion and the client’s reactions. It often involves discomfort, tension, high-pitch laughter, trembling and fidgeting.

These laughter characteristics provide a nuanced understanding of laughter in counseling sessions, helping assess the emotional and relational dynamics between clients and therapists.

#### Session Outcome

The session outcomes were assessed using the Korean version of the Client Satisfaction Questionnaire (K-CSQ). The original CSQ was developed by Larsen et al. [27], while Hwang [19] translated and back translated the CSQ items into Korean. Subsequently, this translation was cross-validated using a confirmatory factor analysis. This study employed an abridged version of the original questionnaire consisting of eight items, known as the K-CSQ-8 [19]. It was rated on a 7-point Likert scale from 1 (*Not at all*) to 7 (*Very much*) with a total score range from 8 to 56. Higher scores indicated greater satisfaction with metaverse counseling. Example items in the questionnaire included statements such as “The client would be content with the level of assistance they received during counseling.” and “The client would have received the type of counseling service they desired.” To allow an objective third-party coder to assess and evaluate counseling satisfaction, the survey questions were modified from self-reporting to being observed by an external observer. Hwang [19] reported a high level of internal consistency reliability, with a coefficient of 0.97 for measuring client satisfaction, and the reliability of session satisfaction assessed by raters in this study was 0.86.

### Data Analysis

The study began by examining the participants’ demographic characteristics, investigating the correlations between variables, and analyzing the frequency of different types of laughter during metaverse counseling. The data collected in this study exhibited a hierarchical structure involving sessions and clients, which created a multilevel structure with repeated measurements and group clusters. To address this issue, a multilevel modeling approach was employed to handle the clustered longitudinal data. For sampling, models that aim to explicitly account for the third level of clustering frequently face challenges when there are fewer than 10 clusters [38]. As there were only seven counselors in the present study and were not nested with enough clients (*M* = 3.71) within each cluster, it was not deemed sufficient for a three-level model. As a result, the therapist effect at level 3 was not measured. All data analyses were performed using IBM SPSS Statistics for Windows, version 27.0. To mitigate the bias stemming from the small sample size and absence of missing data, the restricted maximum likelihood estimation method (REML) was used following the recommendations of Kenward and Roger [22]. When using REML to estimate model parameters, even a relatively small number of clusters as few as 10 with small cluster sizes as low as 5 [30] can yield unbiased estimates of fixed effects [3, 37]. As a group size of average 25 is generally considered small but acceptable number and treated normal in educational research [25], the sample size was deemed sufficient. This was proved again by utilizing a priori power analysis with power of 0.8 (*d* = 0.50). While REML does not fully correct for the downward bias for small sample’s standard errors, the Kenward–Roger approximation was further applied to maintain the nominal Type I error rate in small samples [10, 22]. Additionally, the normal distribution of residuals was validated at both levels, and homoscedasticity [18] was verified by examining residual histograms and a residual scatter plot, which led to confirmed basic multilevel assumptions. The dependent variable was then measured using the scores for session outcomes (session satisfaction) as assessed by the raters. The independent variables included session number (time) and different categories of laughter.

In the initial null model, the intra-class correlation coefficient (ICC; [36]) was computed to gauge the extent to which variances could be attributed to individual differences. This step aims to assess the suitability of multilevel modeling. ICC values generally range from zero to one and indicate the degree of dependency within the nested data structure. Level 1 predictors included session time and laughter categories centered on the group mean for each session. Group mean-centered laughter was calculated by subtracting the average laughter level for each client (Level 2 mean) from the specific value within each session. At Level 2, grand mean centering was performed for predictors related to the aggregated group mean of laughter across sessions. While observing the fixed effects and cross-level interaction terms, random intercepts at the client and counselor levels were integrated into the analysis to account for individual variation.

## Results

We first described the ratios of various types of client laughter characteristics that appeared in metaverse counseling in Table 1. In the metaverse counseling sessions, 25.8% of the laughter were rated cheerful, and 34.0% were rated with polite laughter. Conversely, reflective laughter had the lowest rate (7.8%). Contemptuous laughter was observed at 12.9%, with nervous laughter showing 19.5% of instances.

**Table 1:** Occurrence of Different Types of Laughter

Types of laughter	Occurrence rate
Cheerful	25.8
Polite	34
Reflective	7.8
Contemptuous	12.9
Nervous	19.5

Note. The numbers in the table represent percentages of laughter frequency throughout whole counseling sessions

Table 2 shows the correlations between the variables of interest at both levels. The two levels are categorized as session (Level 1) and client (Level 2). Cheerful, polite, and nervous laughter showed significant positive relationships with session outcomes at the session level (level 1). There was no significant relationship between reflective or contemptuous laughter and session outcomes at the session level (Level 1). However, at the client level (level 2), only polite and reflective laughter showed a significant positive relationship with the session outcomes. Furthermore, a stronger positive correlation between laughter and session outcomes was observed at the client level (level 2) than at the session level (level 1).

**Table 2:** Descriptive Statistics and Intercorrelation between Measures per Session and Client

	*M*	*SD*	1	2	3	4	5	6
Sessions (*n* = 159) Clients (*n* = 26)								
1. Cheerful	2.37	3.86		.378^**^	.079	-.018	.072	.237^**^
2. Polite	3.56	3.04	.537^**^		.102	.078	.364^**^	.245^**^
3. Reflective	0.17	0.45	.078	.338		.036	-.018	.150
4. Contemptuous	0.52	1.29	.008	.456^*^	.095		.477^**^	.055
5. Nervous	2.28	3.73	.118	.496^*^	.080	.628^**^		.250^**^
6. Session Outcome	5.82	0.66	.273	.527^**^	.418^*^	.236	.344	

Note. The session level is above the diagonal line, and the client level is below it.* M* = mean; *SD* = standard deviation; *N* = number

^*^p < .05, ^**^p < .01

**Fig. 1:**
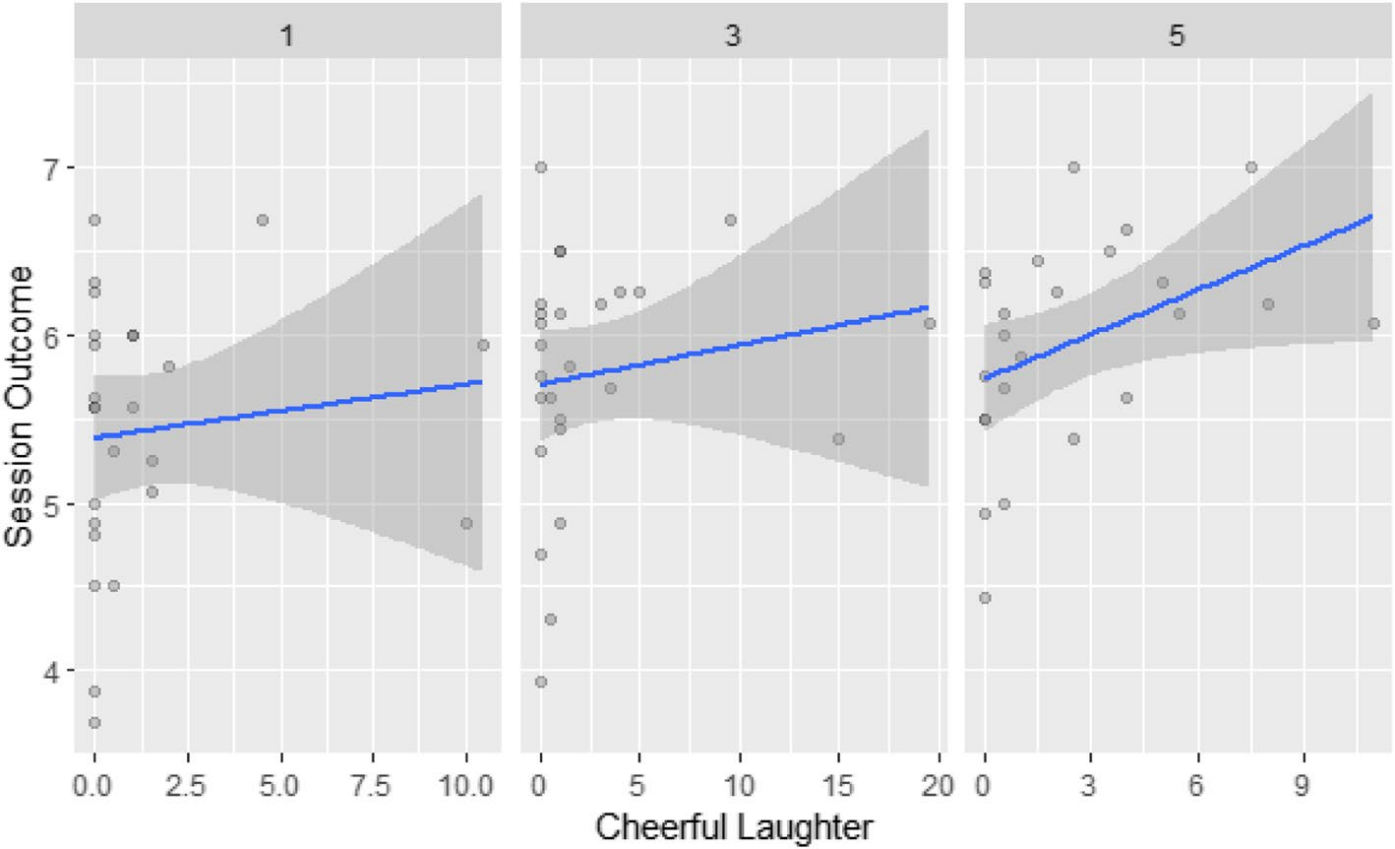
Relationships between Cheerful Laughter and Session Outcomes over the Sessions

Subsequently, we observed fixed and random effects in a multilevel model predicting session outcomes (outcome and dependent variable), divided into five subcategories, into which laughter (predictors and independent variables) was classified. Table 3 shows the results of the multilevel analysis predicting session outcomes. A null model (Model 1) with no level predictors was first used to determine the ICC (0.40) of the session outcome. Inter-individual differences explained 40% of the total variance in session outcomes. Model 2 then presents the added session-level parameters, showing that session outcome (*est.* = 0.06, *p* < 0.05) increased as sessions passed. It also showed that the levels of cheerful laughter (*est.* = 0.04, *p* < 0.05) and nervous laughter (*est.* = 0.04, *p* < 0.05) had a significantly positive relationship with session outcomes at the session level (Level 1). This indicates that cheerful and nervous laughter positively affects the session outcomes. Finally, Model 3 observes the added Level 2 parameters at the client level. Although none of the clients’ laughter significantly affected session outcomes at the client level (Level 2) within laughter categories, cheerful laughter (*est.* = 0.04, *p* < 0.05) only showed a significantly positive relationship with session outcomes at the session level. Finally, a within-level interaction effect between session and cheerful laughter was observed for session outcomes (*est.* = 0.01, *p* < 0.05). The positive relationship between session outcomes and cheerful laughter increased as the sessions proceeded (see Fig. 1). Table 3 presents the random intercepts at the client level in addition to the fixed effects while Table 4 presents the concise model and equations with each parameter added in each level.

**Table 3:** Fixed and Random Effects of Multilevel Models for Session Outcomes

	Model 1			Model 2		Model 3	
**Fixed Effects**	*Est*	*p*	*Est*		*P*	*Est*	*p*
Level 1							
Intercept	5.74 (0.09)	< .001	5.34 (0.12)		< .001	5.18 (0.17)	< .001
Session			0.06 (0.02)		< .05	0.06 (0.02)	< .05
Cheerful _W_			0.04 (0.01)		< .05	0.04 (0.17)	< .05
Polite _W_			0.00 (0.02)		0.87	−0.01 (0.19)	0.73
Reflective _W_			0.13 (0.09)		0.16	0.09 (0.1)	0.37
Contemptuous _W_			−0.03 (0.04)		0.51	−0.03 (0.04)	0.52
Nervous _W_			0.04 (0.02)		< .05	0.04 (0.02)	0.05
Session × Cheerful_W_						0.01 (0.00)	< .05
Level 2							
Cheerful _B_						−0.03 (0.04)	0.38
Polite _B_						0.06 (0.06)	0.34
Reflective _B_						0.59 (0.44)	0.20
Contemptuous _B_						−0.03 (0.20)	0.89
Nervous _B_						−0.01 (0.05)	0.91
**Random Effects**		95% CI			95% CI		95% CI
Residual _Level 1_	0.26	[0.21–0.38]	0.24		[0.18–0.30]	0.24	[0.18–0.30]
Intercept _Level 2_	0.17	[0.08–0.35]	0.12		[0.56–0.26]	0.12	[0.54–0.28]
ICC _Level 2_	0.40		0.33			0.33	
AIC	274.29		280.20			290.95	
BIC	280.33		286.15			296.83	

Note. The AIC and BIC were estimated using REML estimation, and fixed effects were estimated with robust standard errors. _W_, within-client laughter; _B_, between-client laughter

**Table 4:** Models and Equations

Models	Equations
Unconstrained Model (no independent variables)	Level 1: *CSQ*_*ij*_ = *β*_*0j*_ + *r*_*ij*_ Level 2: *β*_*0j*_ = *γ*_*00*_ + *u*_*0j*_
Model 1 (Level 1 independent variables)	Level 1: *CSQ*_*ij*_ = *β*_*0j*_ + *β*_1j_*(*SESS*_*ij*_) + *β*_2j_*(*CHEER*_*ij*_) + *β*_3j_*(*NERV*_*ij*_) + *r*_*ij*_ Level 2: *β*_*0j*_ = *γ*_*00*_ + *u*_*0j*_, *β*_*1j*_ = *γ*_*10,*_* β*_*2j*_ = *γ*_*20,*_* β*_*3j*_ = *γ*_*30*_
Model 2 (Levels 1 and 2 independent variables)	Level 1: *CSQ*_*ij*_ = *β*_*0j*_ + *β*_1j_*(*SESS*_*ij*_) + *β*_2j_*(*CHEER*_*ij*_) + *β*_3j_*(*NERV*_*ij*_) + *r*_*ij*_ Level 2: *β*_*0j*_ = *γ*_*00*_ + *γ*_01_*(C*CHEER*_*j*_*)* + *γ*_02_*(C*NERV*_*j*_*)* + *u*_*0j*_ *β*_*1j*_ = *γ*_*10,*_* β*_*2j*_ = *γ*_*20,*_* β*_*3j*_ = *γ*_*30*_
Model 3 (Levels 1 and 2 independent variables with cross-level interaction)	Level 1: *CSQ*_*ij*_ = *β*_*0j*_ + *β*_1j_*(*SESS*_*ij*_) + *β*_2j_*(*CHEER*_*ij*_) + *β*_3j_*(*NERV*_*ij*_) + *r*_*ij*_ Level 2: *β*_*0j*_ = *γ*_*00*_ + *γ*_01_*(C*CHEER*_*j*_*)* + *γ*_02_*(C*NERV*_*j*_*)* + *u*_*0j*_ *β*_*1j*_ = *γ*_*10*_ + *γ*_11_*(C*CHEER*_*j*_*)* + *γ*_12_*(C*NERV*_*j*_*)* *β*_*2j*_ = *γ*_*20*_ + *γ*_21_*(C*CHEER*_*j*_*)* + *γ*_22_*(C*NERV*_*j*_*)* *β*_*3j*_ = *γ*_*30*_ + *γ*_31_*(C*CHEER*_*j*_*)* + *γ*_32_*(C*NERV*_*j*_*)*

Note. To keep the equations concise, only the parameters for session, cheerfulness and nervousness laughter are included in this table. C refers to the aggregated mean value

## Discussion

This study aimed to reveal the effects of client laughter on metaverse counseling. Among the five distinct characteristics of client laughter, cheerful and polite laughter occurred most frequently in all sessions. However, nervous and contemptuous laughter were less frequently seen, while reflective laughter was rarely seen in metaverse counseling sessions. Furthermore, cheerful laughter and nervous laughter had a positive effect on session outcomes at the session level, while other types of laughter resulted in non-significant results. More specifically, a significant effect of cheerful laughter on session outcomes was observed regardless of the influence of the client’s overall laughter on session outcomes. This implies that the influence of cheerful laughter on session outcomes was considerably significant between sessions. Conversely, nervous laughter did not show any significance in the relationship between overall client laughter and session outcomes. Our findings also showed that as the counseling sessions progressed, cheerful laughter between sessions had a greater impact on session outcomes. This implies that session satisfaction was reinforced, with the influence of cheerful laughter becoming more crucial during the later stages of metaverse counseling.

In the clinical context, laughter describes its meaning in various performances related to the release of psychic energy, stress, anxiety, depression, and interpersonal relationships [4, 6, 24, 35, 45]. Especially in the counseling context, the presence or absence of laughter could support or interrupt therapeutic work and the relationship between the client and the therapist in perceiving genuineness or defensiveness [14]. Therefore, it is important to investigate the specific types of laughter elicited during therapy sessions.

In this study, polite laughter was observed to be the most frequent type of laughter, which is consistent with the findings of Provine [42] and Gupta [14]. However, this study found that polite laughter had no significant effect on session outcomes. This could be explained by differences in the structure and features of cheerful and polite laughter. Previous researchers have suggested that polite laughter is generally triggered by interpersonal relationships and that this laughter can function as a flexible social factor used in early communication situations with others, such as social lubricants [42]. In such situations, polite laughter is used to maintain conversation, loosen or assess the situation softly, or hide nervousness by minimizing the emotions [14, 44]. Meanwhile, Sabonytė [44] proposed evidence of differences between cheerful and polite laughter, examining structure and acoustic features of two types of social laughter. According to this study, polite laughter is considered a one-bout laughter, which comprises one or two syllables shorter in duration than cheerful laughter. In other words, the type of laughter distinguished by laughter tempo with shorter duration and tempo was considered polite. Similarly, in a slightly different context, polite laughter occurred frequently at the beginning of the session. These antecedents might explain why polite laughter can be treated as a factor that occurs consecutively and is captured in social interactions rather than a factor that affects counseling satisfaction.

Empirical research has emphasized the frequency and importance of cheerful laughter in therapeutic processes and outcomes. Falk and Hill [9] specifically uncovered the implications of humorous and cheerful laughter in releasing tension during psychotherapy. Further, it is recognized as a treatment intervention to reduce pain, stress, and anxiety, and positive changes at the biological level have been observed in psychotherapy. Lapierre et al. [26] found that the humor test group prevented a decrease in pain tolerance by eliciting cheerful laughter after watching a ceremony video for 30 min. Similarly, Ko and Youn [24] and Mbiriri [35] suggested that laughter therapy itself helps decrease stress and depression and heal pain or unpleasant feelings. Akimbekov and Razzaque [1] revealed the effects of cheerful laughter during the ongoing stressful period of the COVID-19 pandemic and asserted the importance of creating an environment that accelerates laughter to lessen anxiety and pro-stress factors. The results of our study can be extended in that we observed the role of cheerful laughter by referring to the significant relationship between client laughter and session outcomes. Addressing variables such as a sense of humor or cheerfulness in therapy sessions may suggest positive effects resulting from happiness and may be linked to mitigating the effects of stress [2, 4, 14]. Based on this assumption, cheerful laughter can be expanded to be a major variable for predicting session outcomes.

This study also found a positive relationship between clients’ nervous laughter and session outcomes at the session level. This explanation could be interpreted as freeing negative parts of the self to elicit authentic psychological anxiety through laughter across sessions. Clients using nervous laughter may first experience dissonance between the situational context and their responses [14]. Laughter of nervousness can be interpreted in terms of cognitive incongruity. Martin [34] emphasizes humor duality, indicating similarities between anxiety and laughter responses. Based on this emphasis, Granitsas [13] considered laughter a perspective of nervousness that can generate anxiety and can be stimulated from incongruous happenings by suspicion and confusion. Incongruity, as the root of anxiety, could lead to the treatment of humor as suffering or defensive, and without laughter, it would retain a negative sense of reality [13]. Although Gupta [14] agreed that nervous laughter induces cognitive dissonance, clients can use nervous laughter as an expression to free themselves, which can lead to higher session evaluations. Although nervous laughter is significant for counseling satisfaction between sessions, the method of using nervous laughter may differ among clients. Our study found that nervous laughter did not significantly affect session outcomes when client-level variables were added—it can be assumed that the impact of nervous laughter on session outcomes is not as strong as that of cheerful laughter in metaverse counseling.

Unlike Gupta [14] with in-person therapy, the results of this study indicated that reflective laughter was not significantly related to session outcomes. Gupta [14] insisted that reflective laughter is a prominent factor in non-verbal behaviors when explicating session evaluation. These different results may have been influenced by the therapeutic surroundings (i.e., counseling method), which these two studies demonstrated within different counseling modalities: metaverse and in-person. As the information about the emotional state transmitted when laughing is caught through actual facial expressions, body movements, and sounds [44], it becomes more difficult to capture reflective laughter compared to in-person counseling due to the specific characteristics and limitations of the metaverse counseling modality. Reflective laughter might be described as the genesis of contradictions, such as incongruity in nervous laughter, which is the occurrence of laughter due to the uncanniness of inherent psychological incongruity along with enlightenment gained by comparing it with the incapacitation of others or one’s past [48]. In other words, reflective laughter can tentatively be classified as nervous laughter in a metaverse counseling setting. Moreover, Gupta [14] interpreted client laughter rated highly in terms of reflectiveness as somewhat ruefully amused when they came to new realizations about themselves. Therefore, it can be speculated that reflective laughter might be captured as part of laughter. In this study, reflective laughter was observed at the lowest frequency of 7.8%,therefore, it may be difficult for it to appear as a variable that significantly affects session outcomes.

This study also found that session outcomes improved as metaverse counseling sessions progressed. In other words, the satisfaction level with the counseling sessions increased as the metaverse counseling sessions progressed. Moreover, in the later phases of treatment, cheerful laughter during sessions was linked to stronger session satisfaction. In other words, the more cheerful the laughter in each session, the stronger the relationship between laughter and session outcomes in later stages of counseling. Strong client cheerful laughter signifies the development of a positive client-counselor relationship, referred to as warmth and acceptance, intimacy, and a reduction in emotional distance [31, 39, 40]. The early sessions of metaverse counseling may reflect specificity that the absence of actual contact and practical presence can evoke a sense of alienation, making it more difficult for the counselors to cultivate a reliable relationship with the client quickly [29]. In other words, the effect of cheerful laughter in metaverse counseling could be remarkable in reinforcing session outcomes as sessions proceed toward later phases. Togetherness is another principal aspect of laughter in the development of therapeutic relationships. When two people laugh together rather than alone, it is possible to contribute to an increase in positive emotions by inducing a stronger laughter reaction by building a social scene [26]. Additionally, Marci et al. [33] found that client laughter was shared with therapists to form interpersonal dynamics during sessions. The positive consequences of the shared laughter of therapists and clients in therapeutic relationships can be reflected continuously when they laugh together as the counseling session passes. The way laughter emerges between the client and therapist can become a baseline for the initial establishment of a therapeutic relationship. Furthermore, laughter can be the basis for the client’s transference responses and the emotional bond between the client and the therapist that develops over time.

This study had several limitations that have implications for future research. First, there may be differences in prehensible laughter between the two counseling modalities of metaverse and in-person counseling. Counselors and clients use subtle non-verbal behaviors to recognize important interaction cues, such as body gestures and facial expressions, and immediately discern emotional and relational information [15, 46]. Non-verbal communication can be more severely affected by metaverse counseling in online circumstances. Further, some restrictions might occur in fully adopting Gupta’s [14] laughter classification system, as laughter is likely to be expressed differently in each counseling modality and may cause trouble when implemented under certain circumstances. It is necessary to examine the meaningful aspects of laughter in the context of online counseling, such as metaverse counseling, as the references primarily focus on in-person psychodynamic studies. Considering this, further empirical research should be conducted by clearly comparing metaverse counseling with in-person counseling to investigate specifically how variables that affect counseling satisfaction work depend on different counseling modalities.

Second, there may be differences in the cultural contexts of laughter in Korea. Clients in other cultures may have different characteristics, preferred/denied types, and communication styles regarding laughter [14]. Previous research has argued that specific cultural contexts can shape how emotions and expressions are felt and expressed once formed [28, 47]. Thus, it may be difficult to generalize the relationship between client laughter and session outcomes to other cultures.

Third, it will be necessary to obtain a larger sample size while considering other nested factor effects. Although we achieved an appropriate sample size for using REML and obtained a suitable ICC value, increasing the sample size would enhance the generalizability of the study. This limitation may be attributed to the contextual constraints of the unique medium of metaverse counseling [21]. Yet on the opposite side, this can also be seen as an opportunity to examine the unique aspects of metaverse counseling in greater depth. Additionally, future research could enhance the model by incorporating counselor characteristics as an additional nested level above the client cluster, which would lead to increased sample size. Exploring individual counselor characteristics, which may influence client laughter, could also be valuable.

Finally, all clients opted for voluntary participation in metaverse counseling instead of in-person counseling. Clients who accept metaverse counseling instead of in-person counseling may have higher expectations or motivations for that type of counseling service, which may be related to positive evaluations of counseling sessions [16]. In other words, it was difficult to consider the distinct characteristics of each client who chose metaverse counseling, which may have biased their reported laughter during counseling. A potential explanation for the specificity of clients choosing metaverse counseling might be openness, extraversion, and neuroticism [8], personality factors of those who choose metaverse counseling must be further explored.

Despite these limitations, this study is significant in that it applied Gupta’s laughter classification system to a metaverse counseling environment and investigated the variables that cause therapeutic effects in metaverse counseling, which have not yet been developed. Our findings further suggest that clients’ laughter might reflect a range of laughter characteristics while revealing the specific characterized types of laughter in metaverse counseling. In this vein, this study empirically verified the kind of laughter that should be seen in metaverse counseling to affect counseling satisfaction, further dispensing for the practical viability of metaverse counseling. In summary, the results imply that counselors can derive successful session outcomes from clients in metaverse counseling by paying attention to clients’ laughter and recognizing certain characteristics of it during counseling sessions.

## Data Availability

The data presented in this study are available on request from the corresponding author.
